# Report on American Literature

**Published:** 1851-10

**Authors:** Robert Arthur


### ARTICLE V.

*Report on American Dental Literature. Read before the American Society of Dental Surgeons at their Twelfth Annual Meeting, held in Philadelphia,* 5*th,* 6*th and* 7*th of August,* 1851. By Robert Arthur, D. D. S.

The undersigned was appointed at the meeting of this association, held in Baltimore, in March, 1850, to furnish a report on Dental Literature.

It is known to most of the members present, that this committee, consisting originally of three members, was formed at the annual meeting of 1849. In addition to the special duty of making a report on dental literature, the committee was required to take up also the subject of dental education.

Upon consultation, two difficulties occurred to the members of that committee: they could not but believe that the duties assigned it were more than it could profitably attend to, and there was doubt as to what the society desired in the way of a report on dental literature. For these reasons, nothing was done until the meeting above alluded to was called.

It was then, after some discussion, decided that the committee on dental literature should be exclusive of any other subject, and that its duties should be confined to an examination of American dental literature. For reasons stated at length, at the time, this restriction did not meet the views of the undersigned, and although he consented to perform the duty assigned him by your favor, it was with reluctance, and subsequent regret.

In addition to this restriction, the impression made upon the undersigned from the tenor of the discussion which occurred at the time, was, that the society desired nothing more than a brief descriptive catalogue of such works upon Dental Surgery as were the production of American authors.

Governed by these impressions, the undersigned, your committee, engaged in the examination of such works as came within his reach. These were courteously furnished from the library of professor C. A. Harris, and embrace nearly every thing upon the subject, which has appeared in this country.

The first work published in this country, the first at least within the knowledge of the undersigned, is mentioned in a catalogue at the close of Fitch's Dental Surgery. It is by S. Renners, and was published in 1801. Your committee has not been able to procure a copy.

The first work in the hands of the undersigned bears the following title:

*A Treatise on Dentistry.* Explaining the Diseases of the Teeth and Gums, with the most effectual means of Prevention and Remedy; to which is added Dentition, or Rules to be observed during that Interesting Period. By B. T. Longbothom, Surgeon Dentist. 12mo, pp. 68. Baltimore, 1802:

This is a popular treatise, and the author disclaims any intention of endeavoring to make dentists by means of the contents of his little book, regarding the acquirements necessary to constitute a thorough dentist, such as to make it obligatory upon him to study anatomy, and the general principles of medicine.

With a good deal that is fantastical and erroneous, this little work contains no little which is worthy of note, and some of his practice is very much in accordance with that of the present day.

He proposes the cure of alveolar abscess by laying it open to the bottom, and keeping it open by means of lint dipped in tincture of myrrh, or some stimulating balsam.

For abscesses of the antrum, he preferred making an opening into this cavity through the socket of the canine tooth.

He advises the use of a compress for arresting excessive hemorrhage, after the extraction of teeth, very much in the way it is now applied.

He recommends filling the roots of teeth, when, from any cause it is not thought advisable to extract them.

He mentions having seen sets of teeth retained in the mouth by atmospheric pressure. This method of inserting artificial teeth has been claimed to be of more recent origin. It has been seen, however, by the readers of the American Journal of Dental Science, that the elder Gardette, of Philadelphia, accidentally fell upon this plan of inserting complete sets of teeth, as early as 1800.

Between this little work and the next which was published upon the subject, so far as has come within the knowledge of your committee, there was a long interval. In 1819 was published :

*A Practical Guide to the Management of Teeth.* Comprising a Discovery of the Origin of Caries, or Decay of the Teeth, with its Prevention and Cure. By L. S. Parmly, Dental Professor. 12mo, pp. 198 ; Philadelphia, 1819 :

This, also, is a treatise intended for the public. The author, as the title indicates, advances, what at the time of this publication, was a new theory of the causes of dental caries, which he claims as his own discovery. He attributes caries "to the relics of what we eat or drink, (without regard to quality,) being allowed to accumulate, stagnate and putrefy either in the interstices of the teeth, as is most commonly the case, or else in the indentures on their surfaces, favorable for the lodgment of food."

*Lectures on the Natural History and Management of the Teeth;* the Cause of their Decay ; the Art of Preventing its Accession, and the Various Operations, never Hitherto Suggested, for the Preservation of Diseased Teeth. By L. S. Parmly, Dentist. 8vo, pp. 108. New York, 1821. Second Edition.

This work, by the same author, comprises three lectures, purporting to have been delivered at the Franklin House, Broadway, New York. The subject is treated generally for the benefit of the public, as in the work above noticed.

*An Essay on the Disorders and Treatment of the Teeth.* By Eleazar Parmly, Dentist. Third edition, 12mo, pp. 88. New York, 1822 :

This is also a popular treatise. In its general arrangement it is divided into three parts :

1st, The Growth of the Teeth.

2d, The Diseases of the Teeth.

3d, Operations on the Teeth.

The broad proposition is here laid down, that decay of the teeth is universally caused by the action of external agents; and Dr. P. states, that the teeth are predisposed to caries in consequence of their sometimes being of less dense structure, and less capable of resisting the action of the decomposing matter.

*The Family Dentist.* Containing a Brief Description of the Structure, Formation and Diseases of the Human Teeth. By Josiah F. Flagg, M. D., M. S. S., Surgeon Dentist, 12mo, pp. 82. Boston:

This is a popular treatise on the teeth, the object and scope of which are set forth in the advertisement with which it opens, viz.

"1. To give, in as few words as possible, a clear description of the structure and formation of the teeth, and to bring to view those circumstances connected with their growth, with which it is important for every individual to be acquainted.

"2. To give a brief sketch of the most common diseases to which they are liable, together with such directions relative to their treatment and preservation as shall enable the reader to take the necessary care of his own teeth; and of a parent to pay proper attention to the teeth of his children.

"3. To guard against the injurious practice of ignorant operators; and to remove some of those popular prejudices which prevent many from adopting the only mode of treatment calculated to diminish the liability to disease of these useful and important organs."

*On the Teething of Infants*, and the Complaints to which it may Give Rise. By J. Trenor, M. D., Dentist:

This article appeared in the New York "Journal of Medical and Physical Science," in 1823. Dr. T. denies the truth of the position, that the irritation of teething is caused by the reflex pressure of the forming tooth upon the delicate membranes in consequence of the resistance offered by the gum, and attributes it to the irritation of the mucous membrane of the gum, caused by contact with the enamel, which, devoid as it is of vitality, acts as a foreign body.

*Treatise on the Structure, Diseases and Management of the*

*Human Teeth.* By Eleazar Gidney, Dentist, 12mo, pp. 124. Utica, 1824:

This is a rather more extended treatise than any which was published up to this time. It displays evidences of observation and careful reflection upon the subjects of which it treats.

The author contends, that caries of the teeth is produced by internal, as well as external, causes ; but gives preponderance to the latter. He says, that the nerve may be deprived of vitality by extremes of heat or cold acting upon the external surface of the sound tooth, and that it will then suppurate and make its way through the substance of the dentine.

This is not simply a popular treatise, but goes into the subject in sufficient detail to have deserved the attention of the practitioner of the day when it was published.

*A Physiological Inquiry into the Structure, Organization and Nourishment of the Human Teeth.* By J. Trenor, M. D., Dentist, 8vo, pp. 25. New York, 1828 :

Dr. T., in this carefully considered and well written paper, denies that the teeth possess that high degree of organization now generally ascribed to them ; and although he does not regard them as entirely devoid of vitality, places them lowest amongst the organized tissues. He attributes no other office to the pulp than that of depositing upon its surface layer after layer of bone, by exudation, similar to the process by which the shells of the crustacæa are formed, until it is, eventually, entirely extirpated. The nourishment of the teeth is entirely due, in his estimation, to the peridental membrane, and he regards the extirpation of the pulp as productive of no injury to the tooth, and strongly advocates the practice. Dr. T. mentions, in this paper, that Dr. Hudson, of Philadelphia, introduced this practice many years before, and declares his own knowledge of innumerable cases of this kind, treated by Dr. H. ten, fifteen and twenty years before, which, at the time he wrote, were in a perfect state of preservation, and in every way as useful as any other teeth.

This is a very interesting paper, and well worthy the attention of every student of dental surgery ; and, although I am not prepared to agree with the positions taken by Dr. T., I cannot but regard his paper as a clear, carefully considered and plausible argument.

*Observations on Neuralgia, with Cases.* By J. Trenor, M. D., Dentist, of New York. 8vo, pp. 28:

This paper has no date, but was, evidently, published about the time of that last noticed.

Dr. T. takes the ground, that neuralgia is caused by inflammation of dense structure, in which, as somewhat in odontalgia, the nerves having no room for exhaustion give rise to those extremely painful symptoms which mark this complaint. In accordance with this view of the pathology of the disease, his treatment consists in freely laying open the affected part with the lancet. Although the pain is felt over a considerable region, it may generally be traced to some part very circumscribed in extent. He gives three cases in which the trouble seemed to be caused by the teeth, which, however, were quite healthy; relief was obtained by discovering the spot from which the affection seemed to take origin, and freely laying it open. In these cases the incisions were made inside the mouth, which he advises to be done when it is feasible, in order to avoid leaving a scar in a visible part.

Whether these views are correct or not, it is impossible to read this essay without being impressed with the absolute necessity that the competent practitioner of dental surgery should have a general knowledge of medicine. In one or two of the cases here mentioned, the individuals who were subjects of the disease had been under medical treatment, and were sent to Dr. T. under the supposition that the trouble was caused by a diseased condition of the teeth. Although perfectly healthy, the pain in these cases seemed, by very marked symptoms, so clearly to proceed from the teeth, that the conclusion of every one who had not carefully considered such cases, would probably have led to the sacrifice of important teeth, without affording more than temporary relief.

The first systematic treatise published in this country, intended exclusively for the instruction of those who adopted dental surgery as a profession, appeared in 1829. I have not met with a copy of the first edition, and from the second edition now in my hands, I give the title—

*A System of Dental Surgery.* In Three Parts. I. Dental Surgery as a Science. II. Operative Dental Surgery. III. Pharmacy connected with Dental Surgery. By Samuel Shelden Fitch, M. D., Surgeon Dentist. 8vo, pp. 551. Philadelphia, 1835:

This work is principally a compilation as avowed by the author, in his preface to the first edition, and may be regarded as a *resume* of every thing of value which had been written upon the subject of dental surgery, up to the time of its publication. Some of the writers from whom the materials for the work were drawn, are mentioned by the author. It will be seen that he has availed himself of the labors of the most distinguished writers, upon the subject of which his work treats.

"From the French. Martin, Fouchard, Bourdet, Jourdain, Duval, La Forgue, Delabarre, Le Maire, Baumes, Audibran, Rousseau, &c. &c.

"Latin. Baglivi, Bartholini, and others.

"English. Hunter, Blake, Fox, Murphy, Parmly, Kœcker, Darwin, Rush, Chapman, Horner, Coates, &c. &c."

This work, however, is doubtless quite as familiar to all the members of this association as to your committee, and it would subserve no useful purpose to offer an abstract of it.

*Remarks on the Importance of the Teeth*, on the Diseases of the Teeth and Gums and on the Diseases produced by Diseased Teeth with their Modes of Cure; and Directions for forming Regular and Beautiful Sets of Teeth, and for the Preservation of their Health and Beauty. 8vo, pp. 13. By Francis B. Chewnurg, Dentist, Richmond, Va. 1833:

This is a popular treatise. The title is sufficiently descriptive of its contents.

*The Family Dentist, or a Familiar Treatise on the Art of Securing a Beautiful Set of Teeth.* By Dr. Homer Bostwick, Dentist. 12mo, pp. 100. New York, 1835:

Besides others, mention is made in this popular treatise of works on the teeth by Pleasants and White, copies of which have not come into the hands of your committee. Allusion is also made to,

*Dentologia*; A Poem in Five Cantos. By Solyman Brown, A. M., with Notes by E. Parmly:

This production was subsequently republished in the Library part of the Journal.

*An Inaugural Dissertation on the Physiology and Diseases of the Teeth*, Submitted to the College of Physicians and Surgeons of New York, and Publicly Defended, for the Degree of Doctor of Medicine, April 6th, 1835. By Shearjashub Spooner, Member of the Montreal Medical Society, 8vo, pp. 32, New York, 1835.

*Observations Generales sur l'Importance des Dents.* Par A. L. Plough, Chirurgien Dentiste a la Nouvelle Orleans, 8vo, pp. 8, New Orleans, 1836:

This is an appeal to those having charge of young persons upon the great importance of giving attention to their teeth. The author states, among other things, this deplorable fact, that in visiting the best schools, he had taken occasion to examine the mouths of the pupils, and nearly always found them in most wretched condition, no attention having apparently been paid to their cleanliness. He further states, that he found seventy out of every hundred with diseased teeth, gums affected and breath fetid.

*Guide to Sound Teeth, or a Popular Treatise on the Teeth.* Illustrating the whole Judicious Management of these Organs from Infancy to Old Age; in which the Author will attempt to show that the Teeth of all Persons, which are constitutionally well formed, and who enjoy good Health, may, by proper Management and Care, be Preserved to the end of Life. By Shearjashub Spooner, M. D., pp. 208, New York, 1836:

This is the most systematic and elaborate popular treatise which had been published up to the time of its appearance. It goes into the subject at large, in full detail, with the object of placing in the hands of every one such a knowledge of the growth, structure and diseases of the teeth, as will enable him to understand the causes which produce diseases of the teeth, and be able to make use of the best means of avoiding them.

In this work, Dr. Spooner first made public the fact that arsenious acid would destroy the vitality of the dental pulp.

*A Public Treatise upon the Preservation of the Teeth*, comprising the most Useful Rules for Securing their Whiteness and Beauty, with Observations on the Cause and Prevention and Cure of Caries, and their Effect upon the Health; Intended for Families and the Public Generally. By M. Overfield, pp. 80. Winchester, 1838.

*Dental Hygeia.* A Poem on the Health and Preservation of the Teeth. By Solyman Brown, A. M., Author of Dentologia, &c., with notes, pp. 54. New York, 1838.

*Popular Information on the Subject of Dentistry.* By George E. Hawes, and Charles C. Allen, M. D. Dentists. 12mo, pp. 12. New York, 1838.

*Observations on the Structure, Physiology, Anatomy and Diseases of the Teeth.* In two parts. Part I. By Harvey Burdell, M. D., Honorary Member of the Philadelphia Medical Society, and Member of the Medical Society of the City and County of New York. Part II. By John Burdell, Dentist, with Drawings and Illustrations. 8vo. pp. 96. New York, 1838.

*The Dental Art.* A Practical Treatise on Dental Surgery. By Chapin A. Harris, M. D., Surgeon Dentist. 8vo, pp. 377. Baltimore, 1839.

This is the first edition of a work which is now so well known to every member of this association, that any abstract of it's contents would be a work of supererogation. It was the first entirely original work published in this country for the use of the profession exclusively, and still, after having reached a fourth edition, stands alone. Dr. H. has devoted himself to this work with remarkable energy and industry, and certainly deserves the thanks and regard of the profession for what he has done.

It is just as well here to follow this work through its course. It was first published in a series of numbers, a short time previously to being put into book form, in a weekly literary periodical, published in Baltimore, called the "Monument."* It was then issued, after having been considerably modified by the author, in its present form.

In 1845 a second edition appeared, but so much changed indeed, as to render it almost a new work. The title was then changed to that which it bears at present, viz. "Principles and Practice of Dental Surgery," and was enlarged so as to form a volume of six hundred pages. It was also profusely illustrated. The work in this form was generally acknowledged to be the best practical treatise on Dental Surgery, which had ever appeared in any language, and the edition was soon exhausted.

In 1848, a third edition appeared, it was so much increased in size as to contain seven hundred and fifty pages.

In 1850, the fourth edition now before the profession, was published. Considerable changes, improvements and additions, were made in this edition, which comprises some eight hundred pages.

On the first of June, 1839, was issued a specimen number of the "American Journal of Dental Science." The circular of the publishing committee, E. Parmly, E. Baker, and Solyman Brown, sent it forth with many apparent misgivings as to the success of the experiment, and appealed in strong terms to the more intelligent members of the profession, to come forward to its support. The object in commencing this publication, was to afford, through its means, a vehicle for useful information to the numerous practicing dentists in the United States. The Journal was to consist of forty-eight pages, twenty-four of which, were to be devoted to the republication of standard works on dental theory and practice. It was to be issued monthly.

The need of such a publication was evinced by the promptness with which this effort was encouraged. In the fourth number a list of subscribers, embracing the most eminent names in the profession, was published, showing that there were at that time, one hundred and seventy-four subscribers taking five hundred and eleven copies. This may seem a

*There were only some 30 or 40 pages of the work it published, previously to its appearance in book form. *Eds. Jour.*

small number, but when it is recollected, that this was twelve years ago, when the number of intelligent reading dental surgeons was very small, it must be regarded as evidence of a remarkable general interest in the undertaking.

During the first year of its publication the Journal was conducted under the editorial charge of E. Parmly, of New York, and C. A. Harris, of Baltimore, in which city it was printed. It was issued, with some irregularity, in monthly numbers, at the subscription price of three dollars per annum. At the close of the year it came into possession of this Association, which at that time was organised.

The title was changed to that of the "American Journal and Library of Dental Science;" it was to be issued quarterly instead of monthly, as before, and the subscription price was increased to five dollars. It was placed by the society in the charge of C. A. Harris, of Baltimore, and Solyman Brown, of New York. The editors were to be assisted in their labors by twenty collaborators, whose duty it was to furnish matter for the work and to aid in its circulation.

From that period to August, 1850, the Journal continued to be issued under the auspices of the society, under the charge of several editors, appointed yearly, by the society. At the annual meeting of the association in 1850, it was transferred to Dr. C. A. Harris, of Baltimore, the society relinquishing all control of the Journal, which is now a private enterprise.

The publication of the Journal, is, in the opinion of your Committee, an important event in the history of our profession in this country, and he is satisfied, that it has exercised as great a beneficial influence upon the practice of dental surgery as any other cause within his knowledge. It served, as such a periodical should, to bring about a general acquaintance with the different modes of practice pursued by various practitioners, and, to a great extent, enabled each man to compare his own methods with that of others, and seize upon any improvement which offered.

It may be, that the advantages of a free interchange of ideas between persons employed in the same pursuit, and, particularly, that in which we are engaged, do not impress others as deeply as it does your committee. He is satisfied, that nothing tends more towards its real improvement, and he cannot but believe, that the slow and laboring progress made by our important and useful profession for so many years, is to be attributed to the close, narrow-minded spirit which induced every individual engaged in it, (no, not every one ; for, if this had been so we could not now look, with pride, upon the position it has taken,) in his self-sufficing confidence, which never looked beyond mere pecuniary benefit, to lock up in his small soul all he had thought or done in the pursuit of that vocation into which, by a good Providence, he had been placed, not for his own profit alone, but for the benefit of suffering humanity. And, he is satisfied, that the wonderfully rapid strides with which it has advanced in the last ten years, is to be attributed to a free interchange of views amongst its members. And this spirit has been more fostered and encouraged by this association, in spite of its defects, in spite of the disapprobation and fault-finding of those who were too much influenced by mere personal feeling to give it their hearty support, and endeavor to make it what they might suppose it ought to be, than by any other body of men any where. And it is his determination for the general good that must certainly continue to be effected by it so long as he has health and strength to give to the interests of this movement all the energy he can bring to bear upon it.

The Journal was freely contributed to by most of the intelligent members of the association and others who were not connected with it, and contains many valuable papers upon the theory and practice of dental surgery.

Besides the portion of the Journal devoted to the publication of original articles, a portion, as above indicated, was set apart for the republication of standard works which were not easily attainable and some which were entirely out of print. The following is a list of these works in the order in which they appeared:

*Hunter on the Teeth:* a Treatise on First Dentition. By Baumes. Translated from the French by Thos. E. Bond, Jr., M. D.

*A Treatise on the Diseases of the Mouth.* By J. B. Garrot, M. D. Translated from the French by J. B. Savier, D. D. S., for the American Library of Dental Science; with Notes by the Editors, pp. 100. 1844.

*A New Treatise on the Theory and Practice of Dental Surgery.* By J. Lefoulon, Surgeon Dentist, Paris. Translated from the French by Thos. E. Bond, Jr., M. D., A. M., pp. 125. 1844.

*A Treatise on Second Dentition and the Natural Method of Directing it;* followed by a Summary of Stomatic Semeology. By C. F. Delabarre. Translated from the French, pp. 171. 1845.

*A Critical Inquiry into a Few Facts Connected with the Teeth.* By George Waite, Esq., Surgeon Dentist, Member of the Royal College of Surgeons, Lecturer on the Physiology of the Teeth, Author of Surgeon Dentists' Manual, pp. 23. 1846.

*Anatomy of the Dental System, Human and Comparative.* By P. H. F. R. Blandin, Surgeon of the Hotel Dieu, Associate Professor of the Faculty of Medicine of Paris. Translated from the French by Robert Arthur, D. D. S., pp. 140. 1845.

*Complete Elements of the Science and Art of the Dentist.* By M. Desirabode, Surgeon Dentist to the King, assisted by his Son. Translated from the French, pp. 552. 1847 :

Although published anonymously, this work and that on Second Dentition, by Delabarre, are understood to have been translated by Professor Harris, of the Baltimore College of Dental Surgery.

*An Essay on the Structure and Formation of the Teeth in Man and various Animals.* By Robert Blake, M. D. Revised and corrected with notes, by C. O. Cone, M. D., D. D. S., pp. 128. 1848.

*A Popular Treatise on the Teeth.* Containing a History of the Dental Art, etc., etc., also a Full and Accurate Account of the History of Ether or Letheon, for the Prevention of Pain, with Directions for Use. Designed for the Use of

Families, and as a Manual for the Student and Practicing Dentist. By Mayo G. Smith, Practicing Dentist. Illustrated by Numerous Engravings. Second Edition. 12mo, pp. 316. Boston, 1848:

The same volume contains a Treatise on the Inhalation of Ether, for the prevention of pain.

*The Youth's Dentist, or the Way to have Sound and Beautiful Teeth.* Designed for the more Intelligent orders of Parents and Guardians. By J. R. Duval, Dentist; Member of the Royal Academy of Surgery, &c. Translated and supplied with notes. By J. A. Kinson, Surgeon Dentist, Member of the Royal College of Surgeons, London, &c. pp. 102. 1848.

*A Treatise on the Diseases and Surgical Operations of the Mouth and Parts Adjacent,* with notes of Interesting Cases, Ancient and Modern. Translated from the French of M. Jourdain, Dentist and Member of the College of Surgery, pp. 410. 1849:

This excellent translation, although published anonymously, is understood to have been made by P. H. Austen, M. D., of Baltimore.

A few more works will close the list of such as are in the hands of your committee.

*Preservation of the Teeth.* A Family Guide; being Familiar Observations on their Structure and Diseases; with Practical Illustrations and Engravings, embracing the Modern Improvements in Dentistry. By David K. Hitchcock, Surgeon Dentist. 12mo. pp. 92. Boston, 1840.

*The Anatomy, Physiology, and Pathology of the Human Teeth;* with the Most Approved Methods of Treatment. Including Operations, and the Method of Making and Setting Artificial Teeth; with thirty Plates. By Paul Beck Goddard, M. D., M. A. N. S., M. A. P. S., Demonstrator of Anatomy in the University of Pennsylvania, Lecturer on Anatomy, etc., etc. Aided in the Practical Part by Joseph E. Parker, Dentist. Large Quarto, pp. 227. Philadelphia, 1844.

*The Natural History and Disease of the Human Teeth.* By Joseph Fox, M. R. C. S. L., &c. First American from the third London Edition. Remodeled with an Introduction and Numerous Additions. By Chapin A. Harris, M. D., D. D. S. Illustrated with thirty plates, 8vo, pp. 440. Philadelphia, 1846.

The highly valuable work of Mr. Fox, previously to the publication of this edition, was almost out of the reach of the profession in this country, and could only be obtained at a large cost.

Professor H. has done good service by thus bringing it within reach, and also by his additions, bringing it up to the great improvements which have been made in practical dental surgery, since the time of Mr. Fox.

*A Practical Treatise on the Operations of Surgical and Mechanical Dentistry.* Illustrated with four plates. By Samuel C. Harbert, Surgeon Dentist, 8vo, pp. 206. Philadelphia, 1847.

*Teeth—their Structure, Diseases and Treatment.* Illustrated by Numerous Engravings. By John Burdell, Dentist, 12mo, pp. 72. New York, 1847.

*A Popular Treatise on the Diseases of the Teeth,* including a Description of their Structure and Modes of Treatment; together with the Usual Mode of Inserting Artificial Teeth. By Robert Arthur, D. D. S., duodecimo, pp. 187. Philadelphia, 1846.

*The Principles and Practice of Dentistry, Simplified and Condensed,* for the government of those who value the Health of their Mouths. By R. D. Addington, A. B., D. D. S., 12mo, pp. 46. Richmond, 1848.

*A Dictionary of Dental Science, Biography, Bibliography and Medical Terminology.* By Chapin A. Harris, M. D., D. D. S., Professor of the Principles and Practice of Dental Surgery in the Baltimore College of Dental Surgery, Author of the Principles and Practice of Dental Surgery, etc. etc., 8vo, pp. 780. 1849.

*The Medical Student's Guide in Extracting Teeth,* with Numerous Cases in the Surgical Branch of Dentistry. With Illustrations. 12mo, pp. 76. Philadelphia, 1851.

*A Practical Treatise on Dental Medicine;* being a compendium of Medical Science as Connected with the Study of Dental Surgery. By Thos. E. Bond, A. M., M. D., Professor of Special Pathology in the Baltimore College of Dental Surgery, pp. 307. 1851 :

Dr. Bond, in the publication of this valuable work, has very much added to the many obligations the dental profession is under to him for the warm interest, just appreciation of the merits of dental surgery, as a branch of the science of medicine, and earnest efforts towards its improvement. Supplying, as it does, a desideratum long felt. This work ought to be in the hands of every student of Dental Surgery.

With the publication of this work, the library portion of the Journal was discontinued.

Besides the Journal, other periodicals devoted exclusively to the interests of dental surgery have been, and are still published, all contributing towards the useful object of a freer and and broader interchange of views.

*Stockton's Dental Intelligencer*, was I believe the first of these.

There is no copy in the hands of the committee, and he does not know when it was begun or how long it was continued. It is not now in existence.

*The New York Dental Recorder.* Devoted to the Theory and Practice of Surgical, Medical and Mechanical Dentistry. Edited by Charles C. Allen, M. D., Dentist:

This periodical was commenced in October, 1846. It is published monthly, and has furnished a vehicle for many valuable and useful articles on all the branches of Dental Surgery. It is still successfully continued.

*Dental Register of the West.* Quarterly, was commenced in October, 1847 :

It is published under the auspices of the Mississippi Valley Association of Dental Surgeons. Its publication still continued.

*The Dental News Letter*, quarterly, is published by Jones, White & McCurdy, Philadelphia :

It was begun in October, 1847, and is still published.

*Ether and Chloroform :* Their Employment in Surgery, Dentistry, Midwifery, Therapeutics, etc. By J. F. B. Flagg, M.

D., Surgeon Dentist, Member of the Rhode Island Medical Society. 12mo, pp. 189, Philadelphia, 1851:

The importance of a treatise on this subject, at this time, is felt, and the experience of Dr. F. in the use of anesthetic agents, has well fitted him for the work.

