# Errata

**Published:** 1852-01

**Authors:** 


*Errata.*—On page 61 of last number of the Journal, line 29, for "uncommon," read, common. On page 67, line 17, for "as successful," read, a successful. Page 68, 1st line, for "with shining," read, by ruining.

